# Processing Matters in Nutrient-Matched Laboratory Diets for Mice—Microbiome

**DOI:** 10.3390/ani11030862

**Published:** 2021-03-18

**Authors:** Jasmin Wenderlein, Linda F. Böswald, Sebastian Ulrich, Ellen Kienzle, Klaus Neuhaus, Ilias Lagkouvardos, Christian Zenner, Reinhard K. Straubinger

**Affiliations:** 1 Chair of Bacteriology and Mycology, Institute for Infectious Diseases and Zoonosis, Department of Veterinary Sciences, Faculty of Veterinary Medicine, LMU Munich, Veterinärstr. 13, 80539 Munich, Germany; jasmin.wenderlein@micro.vetmed.uni-muenchen.de (J.W.); ulrich@micro.vetmed.uni-muenchen.de (S.U.); 2 Chair of Animal Nutrition and Dietetics, Department of Veterinary Sciences, Faculty of Veterinary Medicine, LMU Munich, Schönleutenerstr. 8, 85764 Oberschleißheim, Germany; linda.boeswald@lmu.de (L.F.B.); kienzle@tiph.vetmed.uni-muenchen.de (E.K.); 3 Core Facility Microbiome, ZIEL—Institute for Food & Health, Technical University of Munich, Weihenstephaner Berg 3, 85354 Freising, Germany; neuhaus@tum.de (K.N.); ilias.lagkouvardos@tum.de (I.L.); 4 Hellenic Centre for Marine Research (HCMR), Institute of Marine Biology and Aquaculture (IMBBC), 715 00 Heraklion, Greece; 5 Veterinary Immunology Study Group, Department for Veterinary Sciences, Faculty of Veterinary Medicine, LMU Munich, Lena-Christ-Str. 48, 82152 Planegg-Martinsried, Germany; Christian.Zenner@tiph.vetmed.uni-muenchen.de

**Keywords:** feed processing, starch gelatinization, laboratory mouse, diet, intestinal microbiome

## Abstract

**Simple Summary:**

Feed for laboratory mice is available in different physical forms. However, there is insufficient standardization in nutrient composition and physical forms. Results pertaining to energy and nutrient digestibility show that differentially processed feed (pelleted vs. extruded) and even batches from the same provider (pelleted vs. pelleted) differ in starch gelatinization. Here we show that feed processing impacts the mice’s gastrointestinal microbiome. Reproducibility and comparability between experiments with differently processed feeds in laboratory mice should not be taken for granted. Therefore, details on dietary ingredients and feed processing should be specified in studies that include animal experiments.

**Abstract:**

The composition of the microbiome is subject to the host’s diet. In commercial laboratory mouse diets, different physical forms of the same diets are available, containing—according to their labels—identical ingredients and nutrient compositions. However, variations in nutrient composition and starch gelatinization due to production processes and their impact on digestibility have been described. In this study, a total of 48 C57BL/J6 mice were assigned to two equal groups and were fed diets (produced with different processes—extruded vs. pelleted) for eight weeks in two biological replicates. At the end of the experiment, samples were collected from five different gastrointestinal regions, including the stomach, small intestine, cecum, large intestine, and an extracorporeal region (feces), and the microbiome was analyzed with 16S rRNA gene amplicon sequencing. The replicates in both experiments differed significantly in their relative abundances of Muribaculaceae species. Furthermore, the gastrointestinal content of pellet-fed mice contained larger numbers of *Lactobacillus* species. These results indicate that starch gelatinization and ingredient composition significantly influence microbial makeup. In conclusion, different feed processing methods may affect fundamental digestive and metabolic processes, impacting animal experiments and biasing microbiome data.

## 1. Introduction

The intestinal microbiome is defined as the entirety of bacteria, archaea, viruses, fungi, and protozoa that inhabit the gastrointestinal tract of humans or animals [1]. The microbiome is composed of trillions of bacterial cells [2], mostly consisting of facultative and strictly anaerobic bacteria. The most abundant phyla are Firmicutes, Bacteroidetes, Actinobacteria, Verrucomicrobia, and Proteobacteria [3]. In monogastric species, these microorganisms contribute to the hosts’ metabolism by fermentation of prececally indigestible nutrients that reach the large intestine. The main products of fermentation are short-chain fatty acids (SCFA) [4]. The microbiome aids in host metabolism and takes part in its physiological processes. During the development of the host, the microbiome is implicated in the maturation of the immune system [5,6] and the gastrointestinal tract [7,8]. Furthermore, the microbiome protects the host mucosa through opsonization [9,10]. It also influences the development of extraintestinal organs, e.g., the cardiovascular system [11] or the nervous system [12,13]. Shifts in the composition of the microbiota may lead to dysbiosis in the gut, resulting in pathological processes [14] and can lead to gastrointestinal disorders, such as inflammatory bowel disease [15,16] and malignancies [17,18]. The microbiome may even affect disease etiology in other organ systems, such as obesity [19,20] and diabetes [21,22]. Therefore, it is crucial to control and standardize all possible factors influencing the microbiome.

The microbiome composition can be influenced by various factors, such as feeding, cage size, enrichment, group size, and feed composition [23]. Mouse feed consists of different carbohydrates: mono-, di-, tri-, oligo- and polysaccharides. Starch is a plant polysaccharide composed of two polymers of D-glucose: unbranched amylose and highly branched amylopectin [24]. Starch serves as a macronutrient in many foods and is the main glycemic carbohydrate in diet [25,26]. From a nutritional point of view, there are three fractions of starch: rapidly digestible starch (RDS), slowly digestible starch (SDS), and resistant starch (RS) [27]. RDS and SDS form when starch is heated with abundant water. Thereby, the semicrystalline form of starch changes its microstructure during starch gelatinization [28,29]. In a heating process under controlled moisture conditions, called extrusion, the gelatinization of starches occurs only partially [30]. The now disoriented starch structure is more accessible to mammalian digestive enzymes and can thus be digested in the small intestine [30]. Higher digestibility of gelatinized starch has been described for cats and horses [31,32], as well as mice [33]. Resistant starch (RS) cannot be digested enzymatically in the small intestine and undergoes bacterial fermentation in the large intestine [34]. Various studies have already demonstrated that animals fed with RS show a change in their microbiome [35,36,37].

According to current feeding protocols for laboratory mice, a murine diet is available either in pelleted, extruded, semi-pelleted or powdered form. Often, all these physical forms are marketed as identical with the same ingredient and nutrient labeling so that they seem to be exchangeable. Desmarchelier et al. (2013) [38] have described changes in microbiome [3] and body condition of mice when fed either powdered or pelleted diet. Rausch et al. (2016) [23] compared factors contributing to the mouse microbiome’s variation and found that chow provider and chow treatment influence the microbiome variation. The contributing factors of why different physical forms of mouse laboratory diets influence the microbiome have not been identified yet. Studies on digestibility and the composition of nutrients in the feed conducted by Böswald et al. (2021) [33] revealed that extruded and pelleted diets significantly differed in their degree of starch gelatinization, which led to differences in digestibility. This study also showed unexpected differences in diet composition between batches of the same diet form, which also influences digestibility. We aimed to investigate whether these variations in diets may impact the composition of the microbiome. In this study, two groups of mice were fed either with a pelleted or an extruded diet, and the microbial gastrointestinal content in various sampling points was analyzed applying 16S rRNA gene amplicon sequencing.

## 2. Materials and Methods

The ethic commission of the veterinary faculty of the LMU Munich (reference number 169-03-05-2019) approved the use of mice in the proposed experiments. Mice were housed in a specific pathogen-free facility at the faculty of veterinary medicine at the Institute for Infectious Medicine and Zoonosis of the LMU Munich.

### 2.1. Animals and Diets

Forty-eight eight-week-old female C57BL/J6 mice (Envigo, Horst, Netherlands) were housed in individually ventilated cages (504 cm^2^ space) (Techniplast GmbH, Hohenpeißenberg, Germany) in groups of two. The cages contained a bentonite bedding (Silikatstreu, Tigerino Crystals Katzenstreu, Matina GmbH, Munich, Germany) enriched with a polycarbonate mouse shelter (Zoonlab GmbH, Castrop-Rauxel, Germany) and nesting material (Zoonlab GmbH). The cages were cleaned weekly. Feed and water were provided ad libitum. Two trials as biological replicates were conducted. All mice underwent a total experimental period of 56 days consisting of an adaptation to the husbandry facility (day 0 to 20), an adaptation to the individual diet (day 21 to 27), a digestibility trial (day 28 to 47) followed by a one week break to stabilize the microbiome (day 48 to 55). On day 56, all mice were sacrificed by cervical dislocation.

Trial 1. Sixteen mice were allocated randomly to two groups. During the adaptation to the husbandry, all mice were fed the same diet. The diet of both groups was then changed on day 21. Group E1 (*n* = 8) was fed an extruded diet. Group P1 (*n* = 8) was fed the same diet from the same manufacturer in a pelleted form. The degree of starch gelatinization in the diets was 57% in E1 and 17% in P1. All further parameters of the diet can be found in Böswald et al. (2021) [33].

Trial 2. The second trial served as a replicate of trial 1. Accordingly, 32 mice were randomly assigned into two groups. New batches of the same diets by the same provider were used. After adaptation as before, the diets of both groups were changed on day 21. Group E2 (*n* = 16) was fed an extruded diet. Group P2 (*n* = 16) was fed the same diet in a pelleted form. The degree of starch gelatinization was 70% in E2 and 17% in P2. All further parameters of the diet can be found in Böswald et al. (2021) [33].

### 2.2. Post Mortem Sampling

After sacrifice, the mice were weighed and positioned under a laminar flow for dissection. Samples were collected from the digesta of the fundus, the ileum approximately 1 cm before the cecum, from the cecum at the apex, and the colon approximately 1 cm after the cecum. The digesta was transferred into a 2.0 mL tube (Eppendorf AG, Hamburg, Germany) containing 600 µL of stool DNA stabilizer (Invitek Molecular GmbH, Berlin, Germany). Tubes were then stored immediately at −80 °C.

### 2.3. Metagenomic DNA Extraction

Metagenomic DNA was extracted according to Reitmeier et al. (2020) [39]. Metagenomic DNA was extracted from samples thawed on ice. A total of 600 µL of the sample was transferred into a 2.0 mL screw-cap tube prefilled with 0.1 mm silica beads (lysing matrix tubes B, MP Biomedicals GmbH, Eschwege, Germany). Subsequently, 200 µL of 4 M guanidinium thiocyanate (Merck KGaA, Darmstadt, Germany) and 500 µL of 5% *N*-lauroylsarcosine sodium salt (Merck KGaA) were added. In the next step, samples were heated for 60 min in a shaker (70 °C, 700 rpm). Lysis of microbial cells was achieved using a FastPrep-24^®^ (MP Biomedicals GmbH) fitted with a CoolPrep adapter (MP Biomedicals GmbH) filled with dry ice. FastPrep-24^®^ was run on a standard cycle (40 s, 6.5 m/s) three times in total. Next, 15 mg of polyvinylpyrrolidone (Merck KGaA) was added, and then the content was mixed. Then, the samples were centrifuged for three min (15,000× *g*, 4 °C). The supernatant was transferred into a 2.0 mL tube. RNase A at 10 mg/mL (Thermo Fisher Scientific Inc., Dreieich, Germany) was added and incubated for 20 min in a shaker (37 °C, 700 rpm). According to the manufacturer’s instructions, DNA purification was achieved with a NucleoSpin gDNA clean-up kit (Macherey–Nagel GmbH and Co. KG, Düren, Germany). Nucleic acid concentrations were then measured using a NanoDrop (Thermo Fisher Scientific Inc.). If not processed immediately, samples were stored at −20 °C.

The procedure of amplicon libraries preparation (V3–V4 region) and sequencing were described in detail previously [39]. Amplicon purification was performed with the AMPure XP system (Beckmann Coulter, Krefeld, Germany) and sequenced in a paired-end mode (PE300; only using reads of 275 each) with pooled samples containing 20% (vol:vol) PhiX standard library in a MiSeq system (Illumina Inc., San Diego, CA, USA) prepared according to the manufacturer’s instructions.

### 2.4. Data Processing

16S rRNA gene amplicon data were analyzed as described previously [40], with the following changes. Raw reads of both experiments were merged using the NGS toolkit 3.3 (https://pypi.org/project/ngs-toolkit/, accessed on 22 October 2020) and were further processed using the “Integrated Microbial Next-generation sequencing” (IMNGS) pipeline [41] based on UPARSE [42]. Sequences were demultiplexed, trimmed to the first base with a quality score < 20, and paired. Sequences with <300 and >600 nucleotides and paired reads with an expected error > 2 were excluded from the analysis. The remaining reads were trimmed by fifteen nucleotides on each end to prevent analysis of the regions with distorted base composition observed at the start of sequences. The presence of chimeras was tested with UCHIME [43]. Zero-radius operational taxonomic units (zOTUs) were calculated using UNOISE 2 [44] from the Usearch 11 package [45], as implemented in IMNGS [41]. zOTUs with a relative abundance ≥0.25% in at least one sample were kept for further analysis. Taxonomy was assigned at an 80% confidence level with the RDP classifier [46]. Taxonomy and zOTUs tables were refined using SILVA [47] and MEGA X [48]. Rhea was used for further analysis in an R programming environment (R i386 3.6.0, R Foundation for Statistical Computing, Vienna, Austria), as described previously [49]. A detailed description of the analysis and the scripts is available online (https://lagkouvardos.github.io/Rhea//, accessed on 7 May 2019). Prior to normalization, samples of each experiment were assigned to unique zOTU tables to guarantee comparability within experiments. The zOTU tables of all study groups are provided in the Supplementary Material (Tables S1–S5). α-diversity was evaluated based on species richness and Shannon effective diversity [50] as explained in detail in Rhea (see GitHub link). β-diversity was calculated based on generalized UniFrac distances [51]. *p*-values were corrected for multiple comparisons according to the Benjamini–Hochberg method [52]. Only taxa with a prevalence ≥20% (proportion of samples positive for the given taxa) in at least one of the groups and a relative abundance ≥0.25% were considered for statistical testing. All given results were statistically tested with the Wilcoxon rank-sum test, if not stated otherwise. Significant zOTUs were then identified by EzBioCloud’s 16 S rRNA gene-based ID [53]. Data were visualized using either OriginPro, Version 2020 (OriginLab Corporation, Northampton, MA, USA) or Illustrator CS6 Version 16.0.0 (Adobe Inc., San José, CA, USA).

## 3. Results

A total of 8,314,670 sequences were detected within both sequencing runs, with an average of 23,161 sequences (SD = 8259) per sample. The complete zOTU table contains a total of 722 zOTUs.

**Figure 1:**
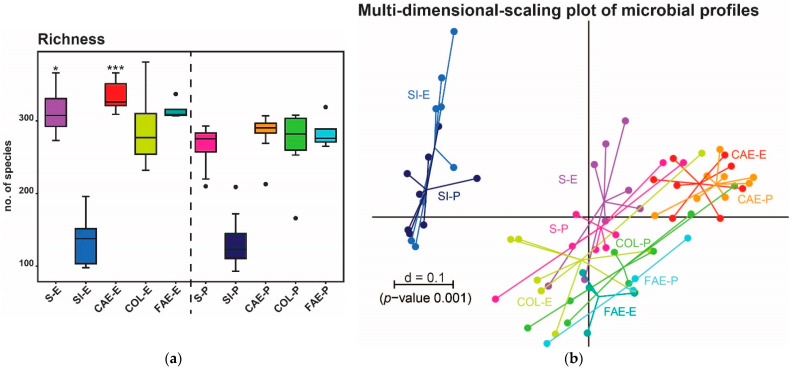
(**a**) α-diversity shown as richness in different intestinal regions of trial 1. The group fed with extruded feed is plotted on the left, the pellet-fed group on the right. Stars indicate significance between the respective gut regions of both groups. (**b**) β-diversity of trial 1 is shown as a multidimensional-scaling (MDS) plot. The scale indicates the distance between samples (d = 0.1 marks 10% difference). E = extruded diet, P = pelleted diet, S = stomach, SI = small intestine, CAE = cecum, COL = colon, FAE = feces. *p*-value summary: * *p* ≤ 0.05, *** *p* ≤ 0.001.

**Figure 2:**
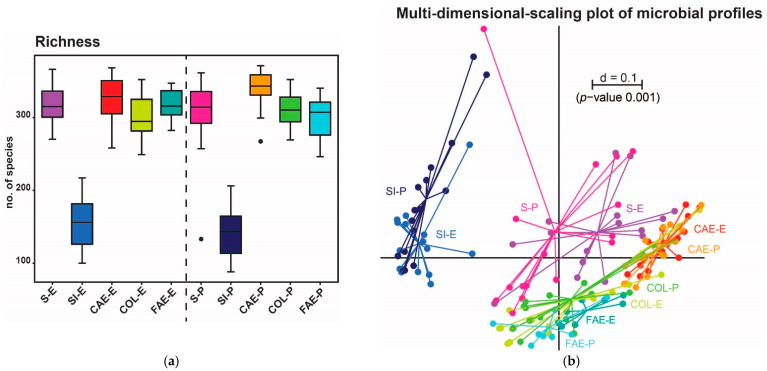
(**a**) α-diversity shown as richness in different intestinal regions of trial 2. The group fed with extruded feed is plotted on the left, the pelleted fed group on the right. (**b**) β-diversity in trial 2 is shown as an MDS plot. The scale indicates the distance between samples (d = 0.1 marks 10% difference). E = extruded diet, P = pelleted diet, S = stomach, SI = small intestine, CAE = cecum, COL = colon, FAE = feces.

For α-diversity analysis, richness was compared in different gut regions, including feces and groups of trial 1 (Table 1, Figure 1a) and trial 2 (Table 2, Figure 2a); these tables also include the sequence read counts of this region and group.

### 3.1. Trial 1

**Figure 3:**
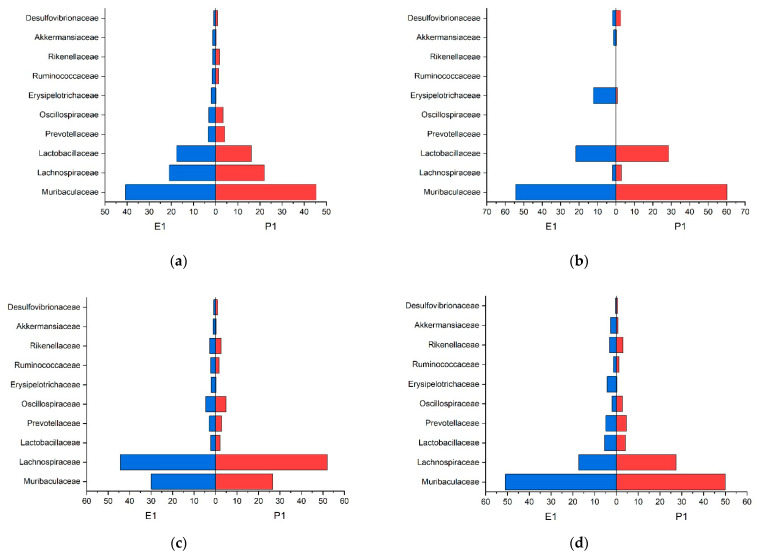
Population pyramid of abundant families of trial 1 given in relative abundance (percent) (**a**) stomach; (**b**) small intestine; (**c**) cecum; (**d**) colon.

In the stomach, small intestinal and colon content, a common core-microbiome consisting of Muribaculaceae, Lachnospiraceae, and Lactobacillaceae, in the previous order descending in numbers, could be defined according to the guidelines of Risely et al. (2019) [54]. The cecal contents’ core-microbiome consisted of the same families, but in the following descending order Lachnospiraceae, Muribaculaceae, and Lactobacillaceae. However, the feces showed different core-microbiota composed of Porphyromonadaceae, Lachnospiraceae, and Prevotellaceae. The relative abundance of these three families within the whole microbiome is displayed in Table 3. The distribution of the families that were higher than 0.5% in relative abundance for trial 1 is presented in Figure 3. As baseline (0 on the x-axis), we used zero percent richness of the according families.

**Figure 4:**
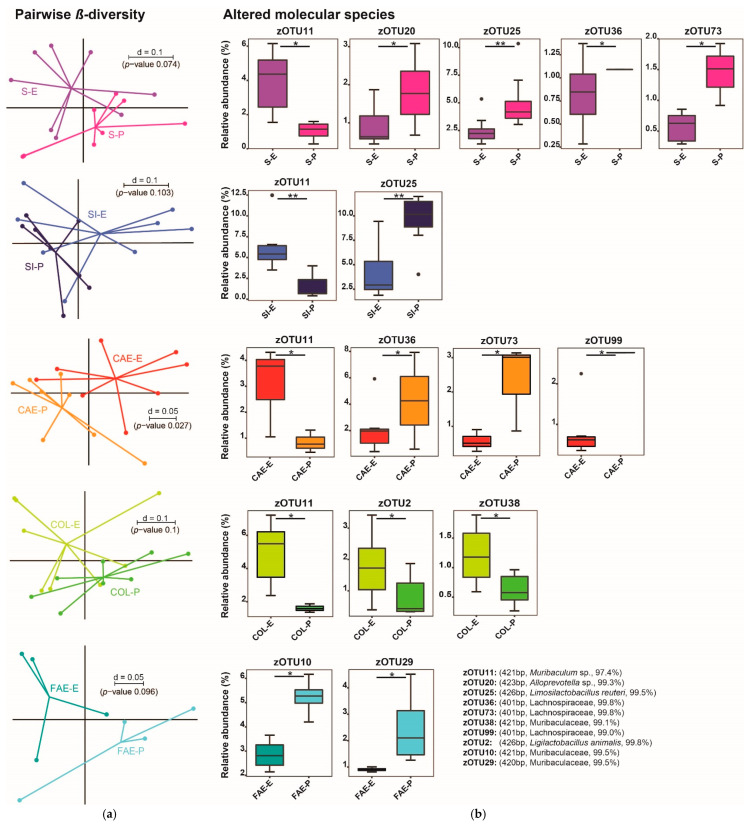
(**a**) Pairwise comparison of β-diversities of trial 1 is shown as MDS plot. Sample origins are indicated by abbreviations. (**b**) Altered molecular species in the respective gut region and feces, displayed horizontally for each gut region and feces according to (**a**). Stars indicate significance between the respective gut regions of both groups. zOTUs were identified by EZBiocloud; the sequence length, the closest relative taxon and the sequence identity score of zOTUs are indicated in the order of appearance. E = extruded diet, P = pelleted diet, S = stomach, SI = small intestine, CAE = cecum, COL = colon, FAE = feces. *p*-value summary: * *p* ≤ 0.05, ** *p* ≤ 0.01.

The β-diversity in the stomachs’ content did not significantly differ between the pelleted and the extruded diet (Figure 4a). By comparing taxa, the genera *Alloprevotella* and *Clostridium* cluster XIVa were significantly more abundant in P1 compared to E1. Looking at molecular species, zOTU 20 (*Alloprevotella* sp.), zOTU 25 (*Limosilactobacillus reuteri*) and zOTU73 (Lachnospiraceae) were significantly more abundant in P1. Significantly more abundant molecular species zOTU 11 (*Muribaculum* sp.) and zOTU 36 (Lachnospiraceae) were detected in E1, the latter being only present in one sample of the pelleted diet but in six samples of the extruded diet (Figure 4b).

In the small intestines’ digesta, the β-diversity did not significantly differ between the two groups (Figure 4a). In a serial group comparison of the taxa, the phylum Actinobacteria were significantly more abundant in E1. The comparison of the zOTUs displayed a *Muribaculum* species (zOTU 11) to be significantly more abundant in E1; however, zOTU 25 (*Limosilactobacillus reuteri*) was significantly more abundant in P1 (Figure 4b).

The cecal content showed a significant difference in β-diversity between the pelleted and the extruded diet (Figure 4a). Taxonomically, the genus *Clostridium* cluster XIVa was more abundant in P1, whereas the taxon Lachnospiraceae incertae sedis was only detected in E1, being significant according to Fisher’s exact test. The molecular species zOTU 11 (*Muribaculum* sp.) was significantly more present in E1. zOTU 36 and zOTU 73 (both Lachnospiraceae) were detected in higher relative abundances in P1 compared to E1. Even though zOTU 99 (Lachnospiraceae) was only present in one P1 sample compared to its presence in six E1 samples, a significant difference was calculated by Fisher’s exact test (Figure 4b).

In the colons’ digesta, the β-diversity did not significantly differ between the two groups (Figure 4a). By comparing taxa, the family Oscillospiraceae was significantly more abundant in E1. At the zOTU level, two molecular species of the family Muribaculaceae (zOTU 11, zOTU 38) were detected that were significantly more abundant in E1. zOTU 2 (*Ligilactobacillus animals*) was also significantly more abundant in E1 (Figure 4b).

The fecal samples displayed no significant difference in β-diversity between the two groups (Figure 4a). Compared taxa also did not show significant differences. When comparing zOTUs, two members of the family Muribaculaceae (zOTU 10, zOTU 29) were significantly more abundant in P1 compared to E1 (Figure 4b).

In the stomach and cecum content, significant differences in α-diversity were detected (Figure 1a). Only minor differences in β-diversity were identified between the compared groups, but analysis at the zOTU level revealed some molecular species with significantly changed abundance. The families Muribaculaceae, Lachnospiraceae, and Lactobacillaceae seem to be most affected by different physical forms of each diet (Figure 4b).

### 3.2. Trial 2

**Figure 5:**
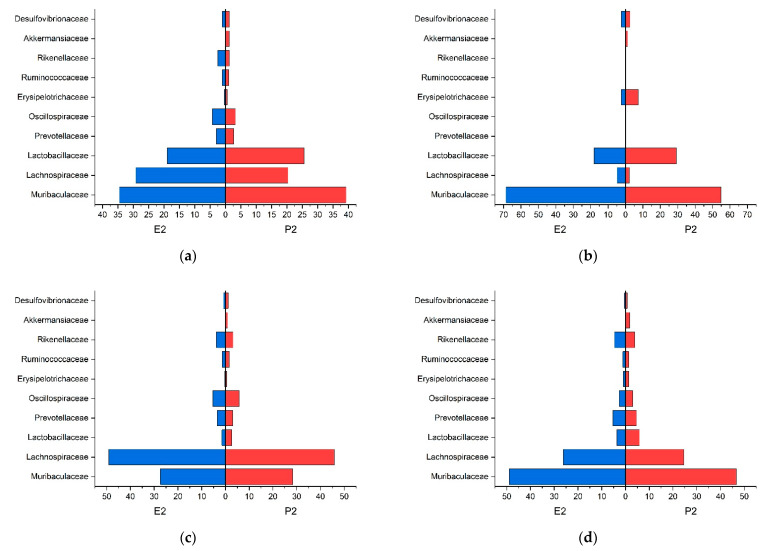
Population pyramid of abundant families of trial 2 given in relative abundance (percent) (**a**) stomach; (**b**) small intestine; (**c**) cecum; (**d**) colon.

A common core-microbiome consisting of Muribaculaceae, Lachnospiraceae, and Lactobacillaceae in the previous order descending in numbers could be defined in the stomach, small intestine, and colon content according to the guidelines of Risely et al. (2019) [54]. In the cecum content, the core-microbiome consisted of the same families Lachnospiraceae, Muribaculaceae, and Lactobacillaceae, in descending order. However, for the feces, we detected different core-microbiota composed of Porphyromonadaceae, Lachnospiraceae, and Prevotellaceae. The relative group strength of the three families on the whole microbiome is displayed in Table 4. The distribution of the families that were higher than 0.5% in relative abundance in trial 2 (Figure 5). As baseline (0 on the x-axis), we used zero percent richness of the according families.

**Figure 6:**
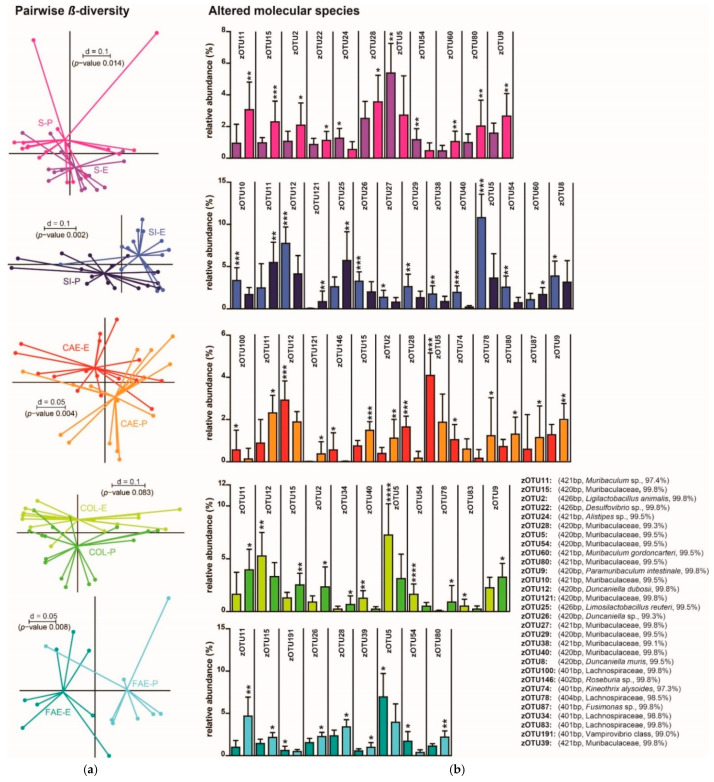
(**a**) Pairwise comparison of β-diversities of trial 2 shown as the multidimensional-scaling plot. Sample origins are indicated by abbreviations. (**b**) Altered molecular species for the respective sample origin, classification and coloring of groups according to (**a**). Stars indicate significance between the respective gut regions of both groups. zOTUs were identified by EZBiocloud; the sequence length, the closest relative taxon and the sequence identity score of zOTUs are indicated in the order of appearance. E = extruded diet, P = pelleted diet, S = stomach, SI = small intestine, CAE = cecum, COL = colon, FAE = feces. *p*-value summary: * *p* ≤ 0.05, ** *p* ≤ 0.01, *** *p* ≤ 0.001, **** *p* ≤ 0.0001.

The gastric samples differed significantly in their β-diversity (Figure 6a). The taxonomic comparison revealed the family Rikenellaceae and its subordinate genus *Alistipes* to be significantly more abundant in E2 compared to P2; the same was true for the genus *Oscillibacter*. In contrast to trial 1, a species of the family Muribaculaceae (zOTU 11) was more abundant in P2 compared to E2. Other members of this family with a similar distribution were zOTU 15, zOTU 28, and zOTU 80. Two more precisely identifiable species, *Muribaculum gordoncarteri* (zOTU 60) and *Paramuribaculum intestinale* (zOTU 9) were also significantly more abundant in P2 than E2. However, some species of the family Muribaculaceae were still significantly more abundant in E2 (zOTU 5, zOTU 54) (Figure 6b).

Pairwise comparisons of β-diversity revealed significant differences between the two groups in small intestinal content (Figure 6a). Taxonomic comparisons showed a significantly higher relative abundance of the phylum Bacteroidetes in E2. In our data, this phylum is mostly composed of the family Muribaculaceae. The phylum Firmicutes was significantly more abundant in P2 compared to E2. However, in this phylum, the family Lachnospiraceae was significantly higher in E2 compared to P2. By evaluating molecular species, we found inverted results to trial 1 for zOTU 11 (*Muribaculum* sp.), as it was now significantly more abundant in P2. Lining up with the stomach content samples of trial 2, zOTU 60 (*Muribaculum gordoncarteri*) was significantly increased in P2. zOTU 121 (Muribaculaceae) was only present in seven samples of P2 but not present in E2; this finding was significant according to the Fisher’s Exact test. However, more species of the family Muribaculaceae were significantly more abundant in E2 (zOTU 10, zOTU 27, zOTU 29, zOTU 38, zOTU 40, zOTU 5, and zOTU 54). Of the family Muribaculaceae, the species *Duncaniella dubosii* (zOTU 12) was significantly more abundant in E2. The same was true for another *Duncaniella* sp. (zOTU 26) and *Duncaniella muris* (zOTU 8). Concordant with trial 1, *Limosilactobacillus reuteri* (zOTU 25), was significantly more abundant in P2 compared to E2 (Figure 6b).

In the digesta of the cecum, the β-diversity did differ significantly between P2 and E2 (Figure 6a). By comparing taxa, we found that the class Bacilli, with its subordinate taxa Lactobacillales, Lactobacillaceae, and *Lactobacillus*, were significantly more abundant in P2 than E2. In an evaluation of molecular species, some species of the family Muribaculaceae (zOTU 11, zOTU 15, zOTU 28 and, zOTU 80) and *Paramuribaculum intestinale* (zOTU 9) were significantly more abundant in P2 compared to E2. Of the same family, zOTU 121 was only present in five samples of P2, but not E2, with a significance according to Fisher’s Exact test. Only zOTU 5 (Muribaculaceae) was significantly stronger in E2; this finding was according to the samples of the stomach and small intestine content of trial 2. In line with the small intestinal content, *Duncaniella dubosii* (zOTU 12) was significantly more abundant in E2 than P2. *Ligilactobacillus animalis* (zOTU2) had a significantly stronger abundance in P2 compared to E2 (Figure 6b).

Pairwise β-diversity did not significantly differ between P2 and E2 for the colon content (Figure 6b). Serial group comparison of this region’s taxa showed no significant differences. Looking at molecular species, some members of the family Muribaculaceae (zOTU 40, zOTU 5, and zOTU 54) and *Duncaniella dubosii* (zOTU 12) were significantly more abundant in E2 than P2; in contrast, zOTU 11 and zOTU 15 (both family Muribaculaceae) and *Paramuribaculum intestinale* (zOTU 9) were significantly higher in P2. *Ligilactobacillus animalis* (zOTU2) had a significantly stronger abundance in P2, in concordance with trial 1 (Figure 6b).

In the fecal samples of trial 2, β-diversity significantly differed between the pelleted and extruded diet (Figure 6b). By comparing taxa, Firmicutes were found to be significantly stronger in E2 than P2. Of this phylum, the class of Clostridia, with its subordinate taxa Lachnospiraceae and *Clostridium* cluster XIVa, all were significantly more abundant in E2. In a comparison of the zOTUs, some species of the family Muribaculaceae (zOTU 11, zOTU 15, zOTU 28, zOTU 39, and zOTU 80) were significantly stronger in P2 than in E2. However, zOTU 5 and zOTU 54, which are other species of the family Muribaculaceae, were found to be significantly more abundant in E2 than P2. Of the same family, zOTU 26, a *Duncaniella* species, was significantly higher in P2 in contrast to the samples of the small intestine of trial 2 (Figure 6b).

Within the second trial, β-diversity differed significantly in almost all gut regions between P2 and E2 but not in the colon (Figure 6a). Interestingly, these differences were not noticeable by just looking at the α-diversity (i.e., richness; Figure 2a). Throughout all observed regions, the family Muribaculaceae seems to be most affected by different physical forms of feed. However, alterations of Muribaculaceae were detected mostly for either P2 or E2, underlining the importance and dominance of this bacterial family in the mouse gut (Figure 6b).

## 4. Discussion

Intestinal content samples (stomach, small intestine, cecum, colon) were collected according to a standardized protocol and displayed consistent results for read counts, normalization and α-diversity (Table 1 and Table 2). In the case of feces, an extracorporeal material, standardization in terms of short, fixed time intervals were not feasible, as they were not removed from the cages immediately after deposition, and environmental factors may have led to further fermentation and reorganization of the microbiome. Therefore, we have not included the feces in all figures and have not considered the feces for further discussion. This observation underlines the importance of standardized sampling protocols and the necessity of fresh feces to obtain valid microbiome data.

Böswald et al. (2021) [33] had described a difference in composition and starch gelatinization for the diets used in this experiment and the feed’s impact on digestibility. Due to the higher degree of starch gelatinization in the extruded diet of trial 1, digestibility of energy and the carbohydrate and fiber fraction was higher in E1 than P1. Diet P2 was a new batch of the diet P1 by the same manufacturer purchased in a 6 month interval. P2 had a much higher starch content and a lower total dietary fiber content than the other three diets. Fiber is the major limiting factor of digestibility in mammalian species. This unexpected but seemingly normal difference in feed composition for different batches resulted in a better digestibility of diet P2 compared to E2 [33].

It is not surprising that these considerable changes in nutrient composition and digestibility affect the microbiome. Therefore, a reproduction of the results of Trail 1 in trial 2 was not possible; changes in the microbiome even between batches of the same diet (P1 vs. P2; Figure 4b and Figure 6b) were observed.

The microbiome in trial 1 showed a stronger relative abundance of several members of the family Muribaculaceae in E1 than P1. *Limosilactobacillus reuteri* was more abundant in P1 than E1 (Figure 4b).

Trial 2 displayed similar results for *Limosilactobacillus reuteri* with a higher relative abundance in the small intestinal content of P2 in comparison to E2. However, the Muribaculaceae family members that were more abundant in trial 1/E1 compared to P1 now showed a significantly higher abundance in P2 compared to E2. Other species of this family, mostly *Duncaniella*, were more abundant in E2 than P2 but have not been significantly altered in trial 1 at all (Figure 4b and Figure 6b).

The effect of diet composition and different physical forms of the feed on the microbiome of mice have been described for caecal content samples in a few studies before [23,38]. Desmarchelier et al. (2013) [38] have described a difference in body weight development when feeding either a pelleted or a powdered diet. Clavel et al. (2014) [3,38] described, based on similar experiments, a pelleted diet to cause an increase in Firmicutes due to more abundant Lachnospiraceae and Ruminococcaceae, and a decrease in Verrucomicrobiaceae due to lower numbers of Akkermansiaceae, compared to powdered diet. The latter authors speculate that differences in feed intake, as well as the shift in the intestinal microbiome, may be associated with differences in the hosts’ weight gain. In the present study, the cecal content of group P1 showed higher numbers of Lachnospiraceae and fewer numbers of Akkermansiaceae compared to E1, verifying the difference in microbiota when different diets forms are consumed. The degree of starch gelatinization seems to be the major determinant for the abundance of Lachnospiraceae and Akkermansiaceae in this case, as the diets E1 and P1 were nutrient-matched in all other aspects (Figure 3c).

Another recently published work from Do et al. (2020) [55] described the effect of a wheat starch diet (WD) in comparison to a gelatinized wheat starch diet (GWD) on the fecal microbiome of mice. They found that the WD diet led to an increase in *Lactobacillus*, *Desulfovibrio* and *Faecalibaculum* and a decrease in the numbers of *Muribaculum* and *Alistipes*. The supply with a GWD diet caused reversed results, displaying an increase in numbers of *Muribaculum* and a decrease in *Lactobacillus* and *Desulfovibrio*.

For the microbiota, we discovered in the colon content of the mice similar results as described by Do et al. (2020) [55], e.g., an increase in Muribaculaceae and a decrease in *Lactobacillus* in the gelatinized diet (E2) and decreased numbers for Muribaculaceae and increased numbers for *Lactobacillus* in P2, the diet with highest total starch content (Figure 5d). Other sampling regions in our experiment did not concord with the previous findings. As Do et al. (2020) [55] were using fecal samples for their study, the findings seem to be comparable to those found in the colons content and feces only. However, Do et al. (2020) [55] did not provide data on the degree of starch gelatinization or dietary fiber in their diets, which makes a comparison with the data presented here difficult.

*Muribaculum* and *Duncaniella* are genera that have been described not long ago [56,57]. Their metabolism is not well characterized, and it is not completely understood how members of these genera may influence the host. Lagkouvardos et al. (2019) [57] described that members of the S24-7 family, now designated Muribaculaceae, have the potential to degrade complex carbohydrates, including host glycans, α-glucans or plant polysaccharides. Besides the carbohydrate metabolic pathway, described metabolic products for this family are vitamin B7 and the amino acid ornithine. Particularly in trial 2, different members of the family Muribaculaceae were found to significantly differ between the pelleted and extruded feed groups, with E2 having more overall counts in the small intestinal and colon content. Several *Duncaniella* species (zOTU 8, zOTU 12, and zOTU 26) were found to be significantly more abundant in the extruded-diet group in trial 2 than in P2. *Duncaniella muris* (zOTU 8 with 99.5% similarity) is described to digest complex plant polysaccharides like hemicelluloses and pectins [57]. zOTU 12 was identified with 99.8% similarity as *Duncaniella dubosii* [58] and could be linked to the *Candidatus* Homeothermaceae strain H5 [59], which is part of the plant polysaccharide guild as well [57]. zOTU 26 has a 99.3% similarity to isolate-110 (HZI) [57] and is described as belonging to the plant polysaccharide guild. This is also true for another species of the family Muribaculaceae, zOTU 29, which has a 99.5% similarity to isolate-036 (Harlan) [57]. For other species (i.e., zOTUs) of our study, an assignment to the species of the study of Lagkouvardos et al. (2019) [57] was not possible.

As Böswald et al. (2021) [33] described, the degree of starch gelatinization is higher in extruded than pelleted diets, resulting in a higher digestibility of similar starch content. Non-gelatinized starch is hardly digestible in the prececal gut so that in mice fed with pellets, a higher percentage of undigested starch is available for microbial fermentation in the large intestine than in mice fed an extruded diet with a similar starch content. Diet E2 contained markedly more total dietary fiber than diet P2, which may be associated with the increase of fiber-fermenting *Duncaniella* in this group. However, *Paramuribaculum intestinale* (zOTU9), found to be more abundant in P2, is described to ferment host glycans [57]. The digestion of host glycans like mucus has already been described for other *Bacteroides* spp. [60]. It is not clear to us why *Paramuribaculum intestinale* is more abundant in the pelleted group of trial 2. A possible reason might be that due to the high digestibility of diet P2 and its low fiber content, less indigestible nutrients are available to the microbiome in the large intestine and thus, species that can ferment host glycans outcompete others. The digestion of intestinal mucus glycans may lead to an erosion of the intestinal mucus barrier. Interestingly, the lack of dietary fiber combined with host glycan degrading bacteria as *Paramuribaculum intestinale* can lead to greater epithelial access for pathogenic bacteria as *Citrobacter rodentium* [61].

With regard to the influence of Muribaculaceae on the host, a decrease in the population strength of *Muribaculum* has been linked to high-fat diets [62] and resistance to obesity in mice [63]. In trial 2, E2 induced a higher relative number of Muribaculaceae in the small intestine, caecum and colon content in comparison to P2. The mice in group P2 showed a significantly higher body fat content and significantly lower body weights than E2 mice (“skinny-fat” phenotype) [33]. This may be linked to an impact of the larger numbers of Muribaculaceae in E2. However, as the exact metabolism of our Muribaculaceae species is a matter of speculation, further research is needed before a definite interpretation is possible.

In addition to changes in the family Muribaculaceae, other species with changed abundance in either experiment, depending on the gastrointestinal region sampled, were identified. One of these species found to be increased in both experiments in the pelleted diet is *Limosilactobacillus reuteri* JCM 1112 (zOTU 25). As an obligate heterofermentative species, *Limosilactobacillus reuteri* JCM 1112 metabolizes glucose primarily through the pentose phosphate pathway resulting in carbon dioxide, acetate, ethanol, and lactate. Another enzyme described for this species is the phosphoenolpyruvate:carbohydrate phosphotransferase system (PTS) subunit IIC for cellobiose and galactitol. It is also able to synthesize vitamin B12 since it possesses *cbi*, *cobi*, and *hem* gene sets [64]. Taken together, *Limosilactobacillus reuteri* could be cross-feeding from other bacteria fermenting starch.

As proposed in other studies [3,23,55] and now verified in this work for pelleted vs. extruded diet of the same ingredients, differences in the processing of commercial laboratory mouse diets led to significant changes in the gastrointestinal microbiome. Unexpectedly, there was even a difference in nutrients content between two batches of diet by the same manufacturer (P1 vs. P2) [33], leading to an increase in the abundance of Muribaculaceae in P2 that has not been observed in P1. Commercial diets for laboratory animals are available in different physical forms and yet marketed as identical products. Due to the variation in feed compositions, obtaining reproducible results between subsequent animal trials was not possible, even when the experiments were conducted in the same research facility and with identical staff as in our project. These changes were surprising since we purchased seemingly identical diets by the same manufacturer only six months after the first trial. Nevertheless, standardization of feedstuff is of utmost importance. The observed effects of a changed microbiome due to different feed forms (e.g., powdered compared to pelleted, extruded and semi-pellet), as in our experiment, have repercussions on mouse experiments published and those to come. Speculating, also the specific preparation of food could possibly impact the human microbiome, an effect which may be considered as a confounder.

## 5. Conclusions

The results of our study show that the processing of laboratory mouse diets significantly influences the development of the microbiome in the animals’ gut. Therefore, researchers should always bear in mind that commercial diets of different batches and preparations will influence the mouse microbiome. Thus, metabolic changes due to this influence need to be taken into account. At this moment, biological reproducibility and a comparison between mouse experiments regarding the microbiome are not given, and future research will be helpful here. Manufacturers should provide more data on the diets´ characteristics. Further, details on the animals’ diets should be given in publications on animal experiments.

## Figures and Tables

**Table 1:** Read count and α-diversity (richness) in trial 1.

Sample		E1	P1	*p*-Value
Stomach content	Read count	23,679	19,682	
	Richness	313.6	264.4	<0.01
Small intestinal content	Read count	21,910	19,252	
	Richness	135.3	134.4	0.92
Cecal content	Read count	22,582	19,614	
	Richness	333.9	278.6	<0.001
Colon content	Read count	21,778	20,216	
	Richness	288.6	270.4	0.98
Feces	Read count	20,236	20,883	
	Richness	315.3	284.0	0.43

E1: mice fed with extruded diet in trial 1; P1: mice fed with pelleted diet in trial 1.

**Table 2:** Read count and α-diversity (richness) in trial 2.

Sample		E2	P2	*p*-Value
Stomach content	Read count	23,127	23,741	
	Richness	320.4	308.1	0.84
Small intestinal content	Read count	22,168	21,752	
	Richness	158.8	146.2	0.36
Cecal content	Read count	24,453	24,077	
	Richness	330.6	342.3	0.41
Colon content	Read count	23,262	22,953	
	Richness	306.0	315.9	0.45
Feces	Read count	21,508	24,614	
	Richness	322.3	302.5	0.37

E2: mice fed with extruded diet in trial 2; P2: mice fed with pelleted diet in trial 2.

**Table 3:** Relative group strength of core microbiome families in trial 1.

Sample	E1 (%)	P1 (%)
Stomach content	79.3	83.5
Small intestinal content	78.1	91.9
Cecal content	76.5	80.9
Colon content	73.8	83.5
Feces	79.3	81.1

E1: mice fed with extruded diet in trial 1; P1: mice fed with pelleted diet in trial 1.

**Table 4:** Relative group strength of core microbiome families in trial 2.

Sample	E2 (%)	P2 (%)
Stomach content	82.6	85.2
Small intestinal content	91.5	86.5
Cecal content	78.0	76.8
Colon content	78.7	77.1
Feces	77.8	75.2

E2: mice fed with extruded diet in trial 2; P2: mice fed with pelleted diet in trial 2.

## Data Availability

The data that support the findings of this study are openly available in the Sequence Read Archive under the reference number PRJNA700529.

## Supplementary Materials

The following are available online at https://www.mdpi.com/2076-2615/11/3/862/s1, Table S1: OTU table stomach, Table S2: OTU table small intestine, Table S3: OTU table cecum, Table S4: OTU table colon, Table S5: OTU Table feces.
